# Inhibition of NLRP3 inflammasome activation by cell-permeable stapled peptides

**DOI:** 10.1038/s41598-019-41211-3

**Published:** 2019-03-20

**Authors:** Arumay Pal, Kurt Neo, Lakshminarayanan Rajamani, Fernando Jose Ferrer, David P. Lane, Chandra S. Verma, Alessandra Mortellaro

**Affiliations:** 1 Bioinformatics Institute (BII), Agency for Science, Technology and Research (A*STAR), 30 Biopolis Street, #07-01 Matrix, Singapore, 138671 Singapore; 2 Singapore Immunology Network (SIgN), Agency for Science, Technology and Research (A*STAR), 8a Biomedical Grove, Singapore, 138648 Singapore; 3 Singapore Eye Research Institute (SERI), The Academia, 20 College Road, Discovery Tower Level 6, Singapore, 169856 Singapore; 4 p53Lab, Agency for Science, Technology and Research (A*STAR), 8A Biomedical Grove, #06-04/05 Neuros/Immunos, Singapore, 138648 Singapore; 5 School of Biological Sciences, Nanyang Technological University, 60 Nanyang Drive, Singapore, 637551 Singapore; 6 Department of Biological Sciences, National University of Singapore, 16 Science Drive 4, Singapore, 117558 Singapore; 7 San Raffaele Telethon Institute for Gene Therapy (SR-Tiget), IRCCS San Raffaele Scientific Institute, Milano, Italy

## Abstract

Interleukin-1β (IL-1β) is a major cytokine that initiates and enhances inflammatory responses. Excessive IL-1β production is a characteristic of most chronic inflammatory diseases, including atherosclerosis, type 2 diabetes, and obesity, which affect a large proportion of the global population. The production of bioactive IL-1β is mediated by a caspase-1-activating complex known as an ‘inflammasome’. The NLRP3 inflammasome has been associated with several human inflammatory and autoimmune diseases and represents a potential therapeutic target for disrupting IL-1β production. We used molecular modeling guided by molecular dynamics simulations to design α-helical stapled peptides targeting the pyrin domain of the adaptor protein ASC to interrupt the development of its filament, which is crucial for NLRP3 inflammasome formation. The peptides were effectively internalized by human monocytic cells and efficiently suppressed the release of the inflammasome-regulated cytokines IL-1β and IL-18, following exogenous activation of the NLRP3 inflammasome. The peptides reduced ASC speck formation and caspase-1 processing thereby suppressing pro-IL-1β processing and release of active IL-1β. This is the first demonstration of the successful use of stapled peptides designed to target the adaptor protein ASC, and can be extended to other inflammatory pathways to disrupt excessive IL-1β production.

## Introduction

Inflammation, resulting from dysregulation of the immune system, is the most common element of age-associated diseases, such as cardiovascular (atherosclerosis), metabolic (obesity and diabetes), and autoimmune (arthritis, colitis) disorders and cancers. The increasing number of patients affected by these disorders highlights an urgent medical need for new and improved treatment options. Chronic inflammation is often associated with excessive production of interleukin-1β (IL-1β)^1^. IL-1β is produced as an inactive precursor molecule (pro-IL-1β) that must undergo maturation before being released into the extracellular environment. Maturation of pro-IL-1β is mediated by caspase-1 activating complexes known as inflammasomes^2^. The core component of the inflammasome is the adaptor protein apoptosis-associated speck-like protein (ASC), which recruits the pro-inflammatory precursors of caspase-1 (pro-casp-1). Several inflammasome configurations have been described, but those containing NLRP3 (NACHT, LRR and PYD domains-containing protein 3) are the best characterized^3^. The NLRP3 inflammasome is highly expressed in innate immune cells, including macrophages, monocytes and dendritic cells^4^. Like most NLRP family members NLRP3 contains a pyrin domain (PYD) at the N-terminal, a central nucleotide-binding domain (NAD) and oligomerization (NACHT) domain, and a C-terminal leucine-rich repeat (LRR). The PYD interacts with the ASC PYD via homotypic PYD–PYD interactions, which allows the caspase recruitment domain (CARD) to recruit pro-casp-1^5^. Under steady-state conditions, NLRP3 inflammasome components are mostly localized to the cytosol and auto-inhibitory mechanisms prevent their assembly. Upon sensing pathogen- and damage-associated molecular patterns, the NLRP3 protein adopts an ‘open’ conformation and recruits ASC to form the inflammasome^6^.

Mutations in *NLRP3* are causative of Cryopyrin-Associated Periodic Syndromes — a heterogeneous group of diseases characterized by spontaneous, periodic inflammation and fever in the absence of overt infection or autoimmune causes^7^. Here, the NLRP3 inflammasome is constitutively active, resulting in augmented IL-1β release from monocytes^8^. The NLRP3 inflammasome has also been linked to chronic inflammation in conditions such as gout^9,10^, pulmonary inflammation^11,12^, atherosclerosis and type 2 diabetes^13–15^.

Most current therapeutic strategies for chronic inflammation target the pro-inflammatory activity of IL-1β: biological anti-IL-1 therapies include the IL-1 receptor antagonist (IL-1Ra) Anakinra, anti-IL-1 receptor and anti-IL-1β antibodies, and IL-1 binding proteins, such as IL-1 Trap. Their efficacy is being assessed in preclinical and clinical studies with promising results^16^. Despite their promise, there is a pressing need for alternative approaches that block inflammasome pathways upstream of IL-1β production, as has been demonstrated recently by specifically targeting the CARD of human ASC by a single domain antibody fragment^17^.

Some endogenous/natural and synthetic small molecules with highly diverse structures can inhibit NLRP3 inflammasome activation and IL-1β release in response to classical NLRP3 activators, such as nigericin, ATP, and urate crystals^18–25^. The underlying mechanisms of action of these molecules are diverse: BHB and glyburide prevent the decline of intracellular K^+^ required for NLRP3 activation and ASC oligomerization^24,25^, whereas parthenolide and Bay 11–7082 inhibit NLRP3 ATPase activity^18^. The mechanism(s) underpinning the effects of MCC950 and MNS are unknown as these molecules do not block K^+^ or Ca^2+^ efflux, or NLRP3–ASC interactions^20,21^. However, some inhibitors have been proven toxic or ineffective in clinical settings, and several potential compounds require further development. Moreover, compounds directly and specifically targeting NLRP3 are still not available. Therefore, developing selective, cell-permeable inhibitors of the NLRP3 inflammasome would be highly valuable.

A new modality that has been gaining interest in the development of specific modulators of protein-protein interactions are peptides; the ability to rationally design high-affinity variants has encouraged their use as potential therapeutics^26,27^. Conformational lability of peptides renders them susceptible to proteolytic digestion, which can be alleviated with the development of macrocyclic bridges that constrain peptide conformations. One such bridge method that has become popular, particularly for stabilizing helical peptide conformations, is known as stapling^28^. Staples consisting of hydrocarbon chains are strategically positioned along a peptide to improve the pharmacological properties of the peptide by increasing target affinity, protease resistance, and in some cases, cellular uptake when compared to unmodified peptides of similar sequences^29^. Critically, a recent study suggested that stapled peptides may offer a significant advantage over small molecules by averting the development of resistance^30^.

Here we designed α-helical stapled peptides that target type-I pyrin interactions to disrupt ASC filament formation. We modeled a set of stapled peptides by combining x-ray structural information of the human NLRP3 PYD (NLRP3^PYD^)^31^, cryo-electron microscopy structural information of the human ASC pyrin domain (ASC^PYD^) filament^32^, and mutational data from the published literature^32,33^. We tuned three physicochemical properties of the peptides to increase their stability and affinity for the negatively charged ASC^PYD^ type-Ia surface by introducing- (i) positively charged residues to enhance the recognition and binding; positive charges are also known to improve cellular uptake, (ii) a large hydrophobic side chain at the center of the peptide to optimize packing inside the hydrophobic pocket on the target surface, and (iii) a hydrocarbon linker (staple) to retain their helical conformation. The resulting peptides were stable, could effectively enter cells and reduced inflammasome-mediated release of IL-1 cytokines from human monocytes in response to NLRP3 inflammasome activation. Furthermore, the peptides disrupted ASC speck formation and casp-1 activation. Our study is the first to report peptide-based modulators as an effective alternative to small molecule inhibitors for disrupting immune-cell function in NLRP3-mediated inflammatory pathways. As the modular organization of NLRP3 and ASC is shared among several other proteins, our findings may serve as a framework for designing peptides that target other innate immune receptors.

## Results

### Determination of the target interface between NLRP3 and ASC

**Figure 1:**
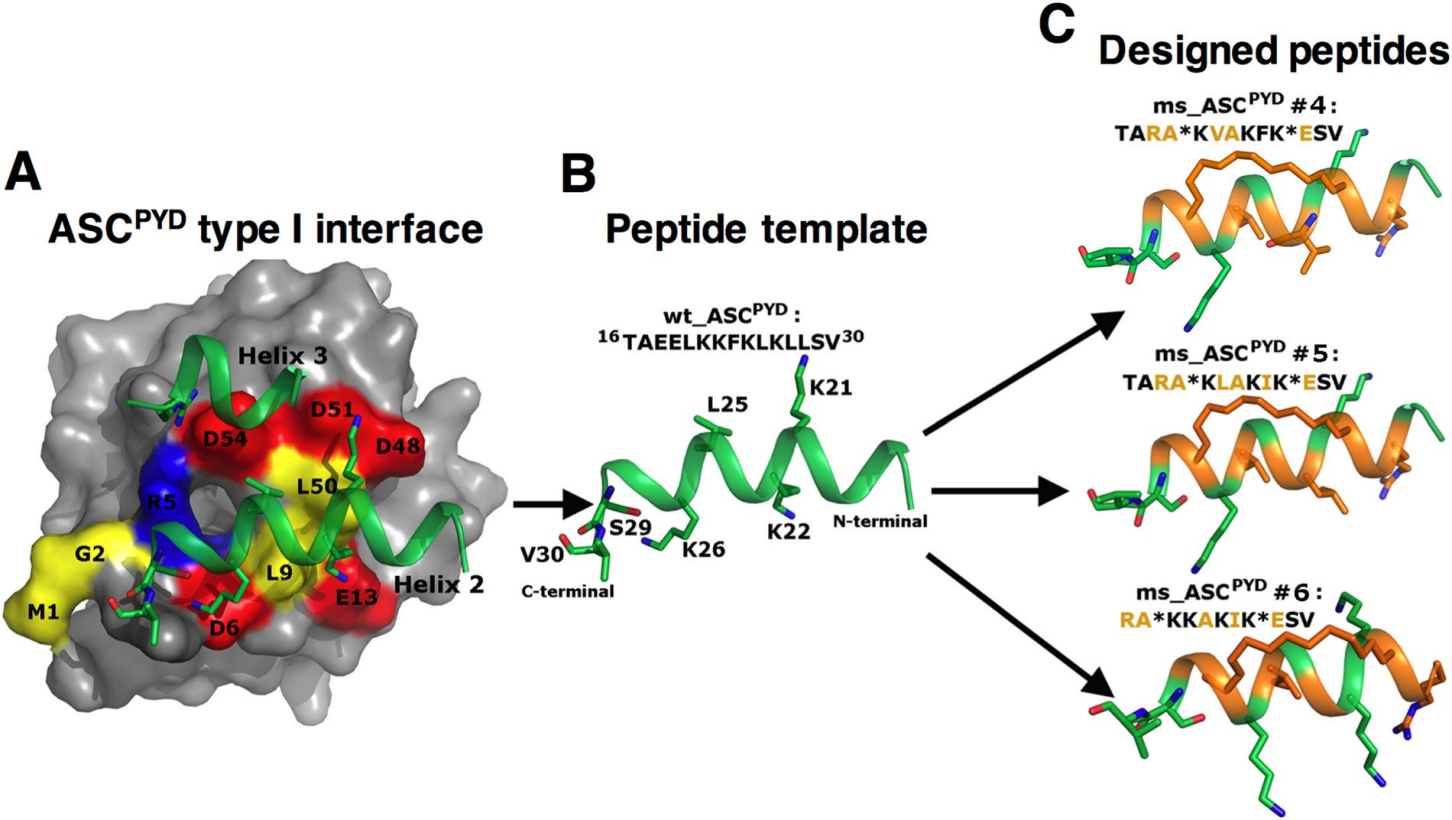
The design process for a set of stapled peptides targeting ASC^PYD^. (**A**) The type-I dimeric interface formed by two ASC^PYD^ monomers is shown, where the negatively charged surface (Type-Ib interface, shown in surface representation) of one monomer interacts with the two positively charged helices 2 and 3 (Type-Ib interface) from the other monomer. Interface residues of the first monomer are marked and colored according to their charges (red = negative, blue = positive, and yellow = hydrophobic). For the second monomer, helices 2 and 3 are in green and sticks depict the interacting residues. (**B**) Sequence and structure of helix 2, the template peptide (wt_ASC^PYD^) used for designing the other peptides is shown in green. Interacting residues are depicted as sticks and are marked. (**C**) Three of the designed stapled peptides are shown with their sequences labeled. The interacting residues are depicted as sticks. The mutation sites are shown in orange. The staple positions are marked as ‘*’ in the sequence, and the ‘hydrocarbon staple’ is depicted as orange sticks.

Given its essential role in forming the inflammasome complex, we chose to target the PYD of the adaptor protein ASC. Similar to other death domain family members, ASC^PYD^ consists of an anti-parallel six helical bundle structure in the Greek key topology. Upon NLRP3 activation, ASC monomers nucleate to form a filament mediated by homotypic PYD–PYD interactions. The human and mouse ASC^PYD^ filament structures solved by cryo-electron microscopy at the resolution of 3.8 Å and 4.0 Å respectively, are the only available structures revealing PYD interactions^32,34^. As the human and mouse ASC^PYD^ structures are identical at the monomeric, tertiary and quaternary levels, we used the human structure (3J63.pdb) for modeling. Within the filament, each ASC monomer engages in three types of interaction (type-I, type-II, and type-III). The type-I interface is the largest of the three, burying ~900Å^2^ of surface area between a negatively charged type-Ia interface of one monomer and a positively charged type-Ib interface from the other (Fig. 1A). The size of this interface is comparable to the average interface area of protein-peptide complexes^35^. Moreover, the ASC^PYD^ type-I interface shares a unique structural feature where helix 3 is shortened, making helix 2 the main contributor to the interactions (engaging ~700 Å^2^ of surface area). All these observations led us to choose the sequence of helix 2 as a template for designing peptides to target the type-Ia interface of ASC^PYD^. Figure 1 outlines the schematic representation of the design process.

### ASC^PYD^ and NLRP3^PYD^ peptide template selection

Examination of the type-I interface (Fig. 1A) suggested that electrostatic interactions have a crucial role in ASC^PYD^ recognition and binding; helices 1 and 4 from the negatively charged type-Ia interface interacts with helices 2 and 3 from the positively charged type-Ib interface, with helix 2 projecting the cationic side chains (Lys21, Lys22, and Lys26) except for Arg41, which is localized in helix 3. Moreover, Leu25 engages in hydrophobic interactions with Leu9 and Leu50 of the type-Ia interface. Considering these factors, we chose residues 16–30 from helix 2 as our peptide template ^16^TAEELKKFKLKLLSV^30^ (Fig. 1B and Table 1). The Leu residues at positions 5 and 12 in this sequence face away from the interface and do not participate in type-I interactions. These two positions were thus chosen to introduce the staple (Fig. 1C). We also noticed that the NLRP3^PYD^ helix 2 sequence ^20^VDLKKFKMHLEDY^32^ was similar to the above template sequence from ASC^PYD^ helix 2, possessing a similar number of positive charges. We therefore also used helix 2 of NLRP3^PYD^ as a peptide template (Table 1).

**Table 1:** Binding energy analysis of the MD simulations of the peptide-ASC^PYD^ complexes.

System	Peptide sequence 1 2 3 4 5 6 7 8 9 10 11 12 13 14 15	ΔGcalc. (kcal/mol)
ASC^PYD^-ASC^PYD^ complex	—	−35.1 ± 11.8
**Peptide template: ASC** ^**PYD**^ **helix 2**		
wt_ASC^PYD^	T A E E L K K F K L K L L S V	−26.5 ± 6.5
wts_ASC^PYD^ #1	T A E E * K K F K L K * L S V	−32.1 ± 6.5
ms_ASC^PYD^ #2	T A R E * K K A K F K * E S V	−35.0 ± 8.2
ms_ASC^PYD^ #3	T A R V * K K A K F K * E S V	−41.9 ± 8.4
ms_ASC^PYD^ #4	T A R A * K V A K F K * E S V	−43.4 ± 7.9
ms_ASC^PYD^ #5	T A R A * K L A K I K * E S V	−43.3 ± 7.4
ms_ASC^PYD^ #6	R A * K K A K I K * E S V	−44.7 ± 7.2
**Peptide template: NLRP3** ^**PYD**^ **helix 2**		
wt_NLRP3^PYD^	V D L K K F K M H L E D Y X X	−13.9 ± 4.2
m_NLRP3^PYD^	V D L K K A K F H L E D Y X X	−24.6 ± 6.1
ms_NLRP3^PYD^	V D * K K A K F H * E D Y X X	−36.9 ± 6.0

For the protein, only the residues making significant contributions to the overall binding energies are reported. The binding energy for the ASC^PYD^-ASC^PYD^ complex (type-I interface) is the first entry for reference. All the designed peptides show much stronger binding energies than the wild-type templates.

Binding energy ΔGcalc was calculated by the MM-GBSA method (Miller *et al*.^57^). The symbol ‘*’ signifies positions that are linked by the hydrocarbon staple; mutated residues are underlined; wt, wild-type helix 2 sequence; m, mutated helix 2 sequence; s, stapled. The N-terminus of each peptide was acetylated (ACE) and the C-terminus was amidated (NHE).

### Docking simulations confirm that the designed peptide binds to the target binding site

**Figure 2:**
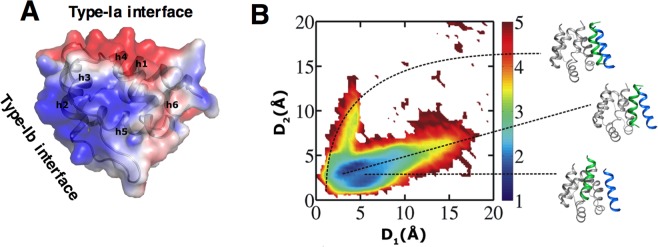
Non-specific electrostatic attractions form the predominant interactions between ASC^PYD^ and the peptides. (**A**) The solvent-accessible surface of ASC^PYD^ monomer (3J63.pdb) colored according to the electrostatic potential (red = negative, blue = positive) shows the complementary charge between the two surfaces. Residues interacting at the homotypic PYD/PYD type-I interfaces are localized at those two clusters. Electrostatic potentials were calculated using the APBS program (http://nbcr-222.ucsd.edu/pdb2pqr_2.1.1) with default parameters. (**B**) Predicted conformational ensemble for ms_ASC^PYD^ #5:ASC^PYD^ complex from coarse-grained docking simulations that utilize long-range electrostatic potentials. Predictive simulations were started by placing a free peptide at different positions away from the protein. The simulated conformations were compared to the type-I ASC^PYD^ interface structure (Fig. 1A) to judge their binding accuracy. The similarity measure (D_1_ and D_2_) quantifies the structural similarity between the predicted peptide-protein binding interface in the docking simulation and the type-I ASC^PYD^ interface structure, where a lower value of D_1_ or D_2_ corresponds to a higher degree of similarity (see Methods). The colored bar indicates the free energy of different binding conformations of the predicted peptide-protein complex. Representative docked peptide conformations (green helix) from three regions of the map are shown in the right panel. ASC^PYD^ is shown in gray. The position of helix 2 (blue helix) in the type-I interface is also shown to judge the binding accuracy of the docked peptide. Close positioning of the green helix to the blue helix represents a predicted conformation (top) similar to the experimental structure. Furthermore, the highest population (blue regions) is found closer to min(D_1_, D_2_) on the map which indicates that peptides docked at the correct binding site in the type-I interface.

To explore whether charge complementation between the peptide (cationic) and the ASC^PYD^ binding surface (type-Ia, anionic, Fig. 2A) was sufficient for site-specific binding of the peptides, we performed a docking search using simulations of coarse-grained models of the protein and peptide where their interaction was modeled solely by electrostatic interactions between the charged residues. Coarse-grained simulations access time scales that are long enough to achieve the sampling of the binding process, which are not yet possible by all-atom simulations. Simulations were started by placing the representative peptide (ms_ASC^PYD^ #5, Table 1) ~35 Å away from the protein (See Methods). As the numbers and the positions of the positively charged residues were similar in all the peptides, we assumed that all other peptides would show similar binding behavior in the coarse-grained simulations. The peptides congregated at the ASC^PYD^ type-Ia binding surface, and their conformations matched the all-atom initial model (Fig. 2B), thus demonstrating that electrostatics is indeed the primary driver of recognition and the designed peptides should successfully target the ASC^PYD^ type-Ia interface. Furthermore, docking simulations between ASC^CARD^ and ms_ASC^PYD^ #5 clearly showed that the peptide does not bind to ASC^CARD^.

### Design, mutation, and characterization of I, I + 7 hydrocarbon-stapled peptide inhibitors

**Figure 3:**
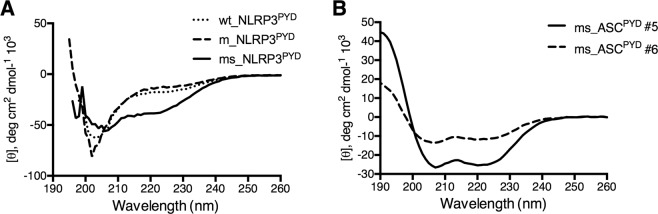
Circular dichroism (CD) spectra of (**A**) NLRP3^PYD^ and (**B**) ASC^PYD^ peptides to measure secondary structures. CD spectra were measured at room temperature in water at a peptide concentration of 100 μM. ms_ASC^PYD^ #5 and ms_ASC^PYD^ #6 showed typical wavelength minima for α-helices at 208 nm and 222 nm, whereas ms_NLRP3^PYD^ showed deviation from a helical trend.

The conformation of helix 2 (^16^TAEELKKFKLKLLSV^30^) bound to ASC^PYD^ (Fig. 1A) was used as the starting structure for the peptide-protein interaction modeling. Following I,I + 7 stapling scheme, we inserted a hydrocarbon staple across positions 5 and 12 along the peptide and then introduced mutations to produce a set of stapled peptides. Two variants of the template peptide were designed – a short version containing 13 residues (ms_ASC^PYD^ #6) and a long version containing 15 residues (ms_ASC^PYD^ #1–5) with two additional residues at the N-terminus (Table 1). These two additional residues were inserted to increase the length of the peptide, thus potentially enabling sufficient conformational flexibility for the region involving the critical Arg3 to remain helical. A similar approach was taken to introduce the staple in the ms_NLRP3^PYD^ peptide. As determined from Circular Dichroism (CD) spectra, inserting the I,I + 7 staple had the intended effect of increasing helicity of the peptides (Fig. 3). The wild-type unstapled NLRP3^PYD^ peptide showed no helicity, which was increased to 48% when stapling was introduced. Percentage of helicity was much higher for ASC^PYD^ stapled peptides #5 and #6, 95% and 70% respectively at 100 μM peptide concentration. Percentage of helicity for the peptides were estimated from the web server K2D3^36^ using CD data.

**Figure 4:**
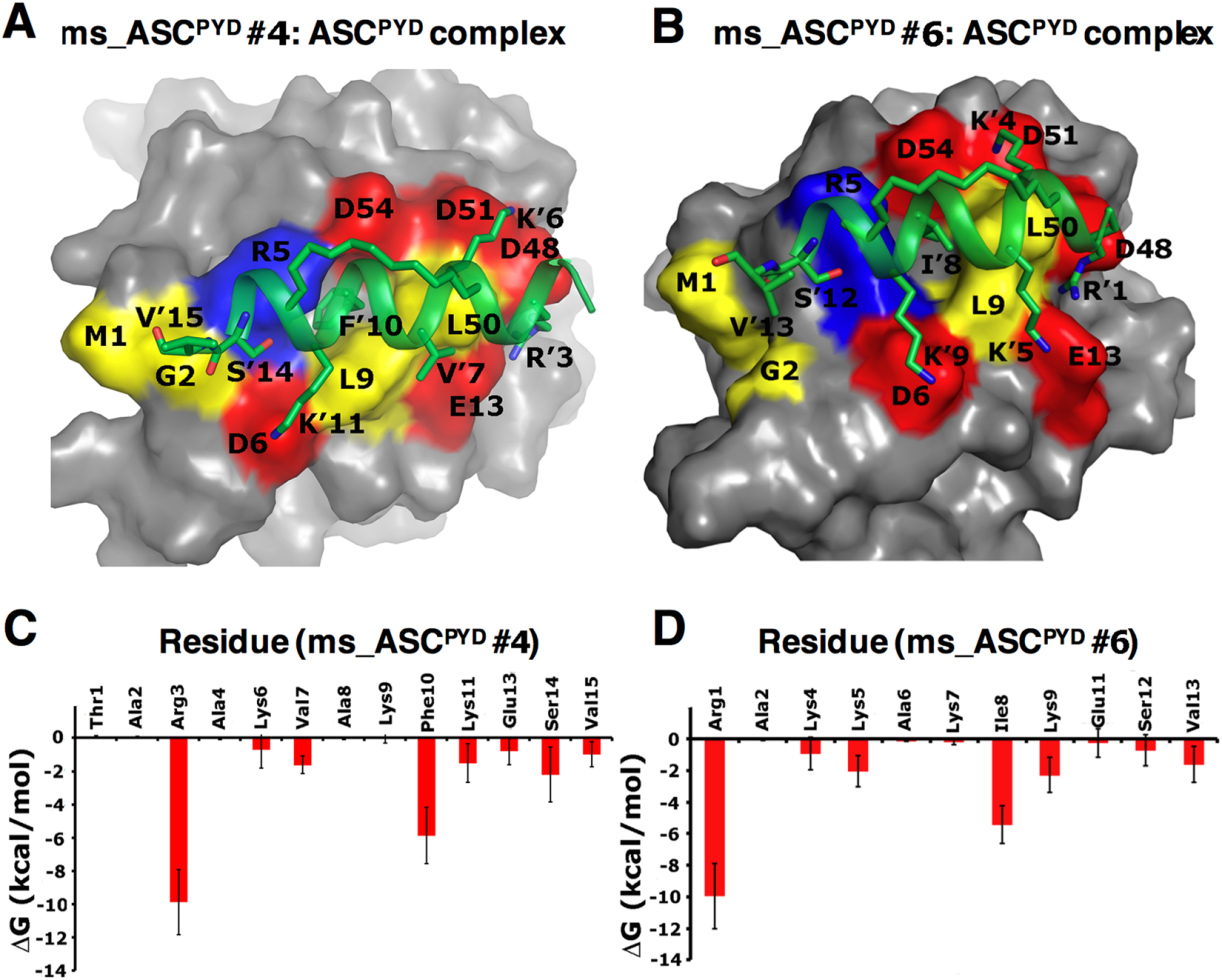
Binding interactions of the designed stapled peptides (green helix) with ASC^PYD^ (gray surface) determined from all-atom MD simulations. Two representative peptides ms_ASC^PYD^ #4 (**A**) and ms_ASC^PYD^ #6 (**B**) are shown. The structures are taken from the end of the 200 ns simulations. Interface residues of the ASC^PYD^ are marked and colored according to their charges (red = negative, blue = positive, and yellow = hydrophobic). Peptide residues are marked with a prime symbol (´). At the peptide N-terminus, the Arg residue (R’3 or R’1) is involved in stable salt bridge interactions with both Glu13 and Asp48 of ASC^PYD^. Two Lys residues (K’6 or K’4; K’11 or K’9) form frequent salt-bridges with residues Asp51, Asp54 and Asp6 of ASC^PYD^ (see Fig. S1). Phe10 or Ile8 (F’10 or I’8) from the central parts of the peptides are involved in hydrophobic contacts with the side chains of Leu9, Leu50, and Arg5 of ASC^PYD^ (details in Fig. 5 and S2). Non-specific hydrogen bonds were observed in the simulations between the main-chain of Ser or Val (S’14 or S’12; V’15 or V’13) from the peptide C-terminal and the main-chain of Met1, Gly2 or Arg5 from ASC^PYD^. (**C** and **D**) Energetic analysis of the MD simulations of the stapled peptide:ASC^PYD^ complex for two representative peptides ms_ASC^PYD^ #4 (**C**) and ms_ASC^PYD^ #6. (**D**) Residue-wise binding energy contributions are shown.

**Figure 5:**
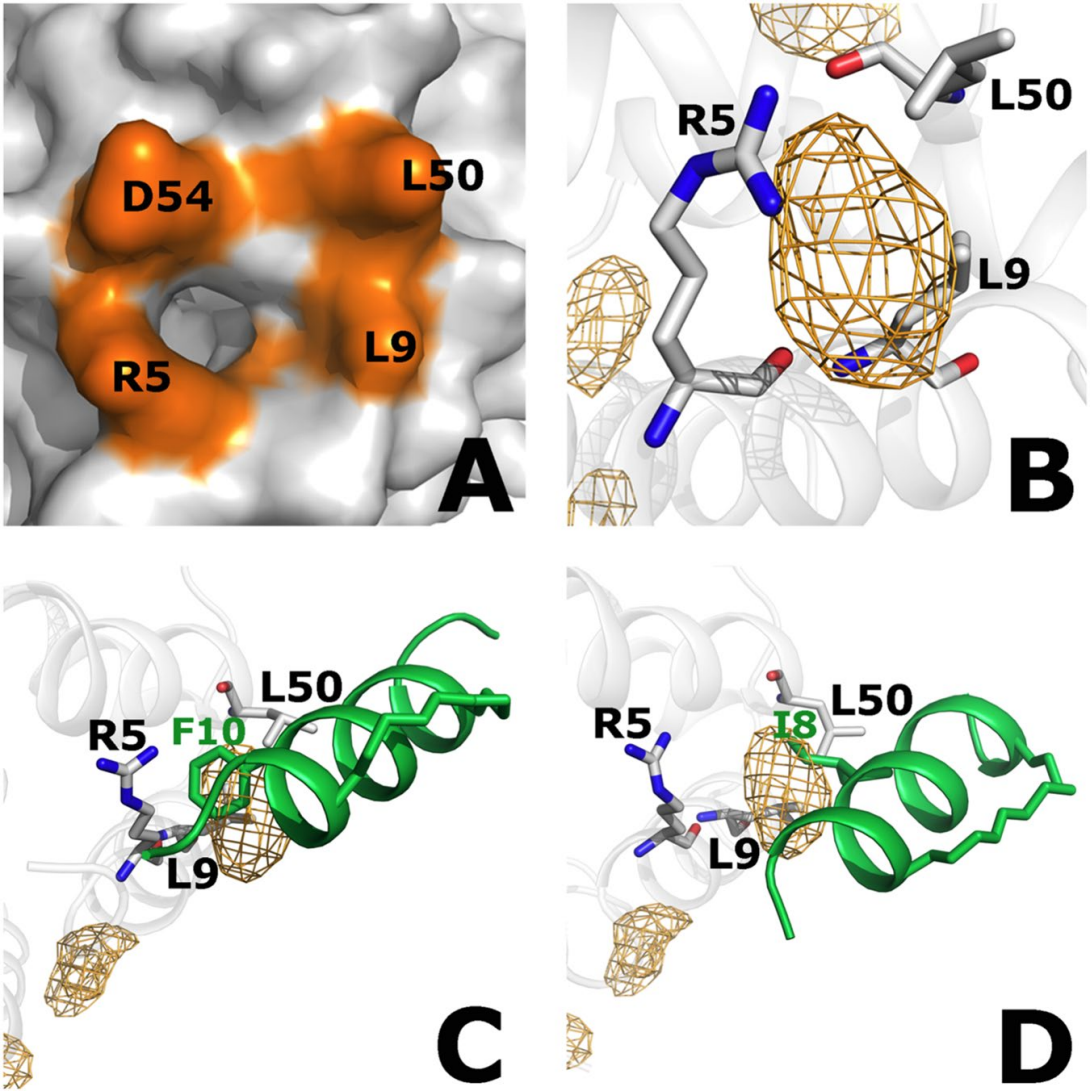
Hydrophobic interactions anchor the peptide at the ASC^PYD^ surface. (**A**) The small hydrophobic pocket between residues Leu9, Leu50 and Arg5 is observed at the ASC^PYD^ type-I binding interface in the experimental structure (3J63.pdb). (**B**) Presence of the pocket was confirmed by benzene-mapping simulation of the ASC^PYD^ monomer, where benzene probes sampled the region (orange grid). The benzene probability grids were generated by using the PTRAJ module of AMBER12 to bin carbon atoms of benzene molecules into 1 Å × 1 Å × 1 Å grid cells from a benzene-mapping MD trajectory. For the visualization of benzene occupancy in the grids, the cutoff isocontour value used was five times the threshold bulk value, which was defined as the highest isovalue at which benzenes were detected in the bulk solvent. This was chosen to filter out most of the spurious binding sites, and highlight those that are likely to be legitimate. (**C**) Phe10 of the peptide ms_ASC^PYD^ #4 fits into the hydrophobic mesh when the benzene-mapped simulated structure of ASC^PYD^ with the hydrophobic mesh is superposed onto the simulated structure of the ms_ASC^PYD^ #4:ASC^PYD^ complex. (**D**) Ile8 of the peptide ms_ASC^PYD^ #6 also fits well into the hydrophobic mesh. The hydrophobic interaction shown markedly contributes to the net binding energy (Fig. 4).

To optimize peptide stability and binding, we introduced up to six further mutations into the wild-type sequence (Table 1). Binding energy contributions for the type-Ib interface residues was calculated from simulations of the ASC^PYD^:ASC^PYD^ type-I complex structure (prepared from 3J63.pdb), which identified the most appropriate residue positions to be mutated (Table S1). At the N terminus, we mutated Glu3 and Glu4 to Arg and Ala, respectively to gain a positive charge and further stabilize the electrostatic interactions and van der Waals contacts of the peptide with ASC^PYD^ (Fig. 4 and Table S2). The Phe8 side chain faces away from ASC^PYD^ and does not interact with the protein; it was mutated to Ala to reduce thermodynamic penalty due to solvating the hydrophobic side chain. We mutated the Leu10 side chain (that inserts into a hydrophobic pocket on ASC^PYD^) to Phe or Ile to increase the hydrophobic packing (Figs 4 and 5). We also mutated Lys7 to Leu in ms_ASC^PYD^ #5, to improve packing against the residues of the hydrophobic pocket. At the C-terminus, we mutated Leu13 to Glu to introduce polar interactions with Arg5 of ASC^PYD^. Although Lys9 faces away from the interface and does not interact with the protein, we maintained this site to gain an extra positive charge, which should aid cell permeability. Using a similar approach, we mutated two positions in the ms_NLRP3^PYD^ peptide to improve the binding energy (Table 1). We noted that all the ASC^PYD^ stapled peptides showed more favorable binding energies compared to the NLRP3^PYD^ stapled peptide, possibly due to less helicity and a missing positive charge at position 11 for the later peptide.

### Validation of peptide binding stability by molecular dynamics (MD) simulations

All-atom MD simulations of the stapled peptide:ASC^PYD^ complexes suggested that all peptides bound similarly to the interactions seen in the type-I interface of the ASC^PYD^ filament (Figs 1A and 4). The Arg1, Lys4, Lys5, Ile8, Lys9, Ser12, and Val13 residues of the short peptide ms_ASC^PYD^ #6 (corresponding residues from the ms_ASC^PYD^ #5 peptide are Arg3, Lys6, Leu7, Ile10, Lys11, Ser14, and Val15) made a significant energetic contribution to their interactions with ASC^PYD^ (Fig. 4 and Table S2). Arg1/Arg3 is involved in stable salt-bridge interactions with both Glu13_t_ (t denotes residues from the target protein ASC^PYD^) and Asp48_t_ (Fig. S1), and in van der Waals interactions with Leu50_t_, thus contributing the highest binding energy among all the peptide residues. This effect helps anchor the peptide N-terminal to the protein surface. Lys4/Lys6 (corresponds to ms_ASC^PYD^ #6 and #5, respectively) forms less stable salt-bridges with one of the negative side chains of Asp51_t_ or Asp54_t_ from helix 4, compared to a more stable salt-bridge between Lys9/Lys11 and Asp6_t_ of helix 1 (Fig. S1). In the case of the shorter peptide ms_ASC^PYD^ #6, an additional electrostatic interaction was observed between Lys5 and Glu13_t_ (Fig. S1). At the C-terminus, non-specific hydrogen bonds were observed in the simulations between the main-chain of Ser12/Ser14 or Val13/Val15 and the main-chain of Met1_t_, Gly2_t_ or Arg5_t_. Another major binding energy contribution comes from Ile8/Ile10 where it engages in van der Waals contacts with a hydrophobic pocket formed on the ASC^PYD^ surface. This pocket was observed at the middle of the type-I interface in the ASC^PYD^ filament structure (Fig. 5A) where it is formed by the solvent-exposed side chains of Leu9 and Leu50 from the type-Ia interface into which Leu25 from the type-Ib interface interacts (Fig. 1A).

To investigate whether this hydrophobic binding pocket on the ASC^PYD^ surface is flexible and can accommodate larger hydrophobic side-chains such as Ile or Phe, an in-house ‘ligand-mapping’ approach was used where benzene probes were used to tease out cryptic regions of the protein, as previously described^37^. The resulting MD trajectories of the ASC^PYD^ monomer structure showed high benzene occupancy in this pocket, indicating that this hydrophobic pocket is exposed in the absence of its binding partner (Fig. 5B). Indeed, the Ile8/Ile10 side chain was well placed (Phe10 in the case of the peptide ms_ASC^PYD^ #4) in the area occupied by the benzenes (Fig. 5C,D); however, the pocket was not large enough to accommodate bigger side chains such as Trp. In the designed peptides, Ile8/Ile10 (in ms_ASC^PYD^ #6/ ms_ASC^PYD^ #5), and Phe10 (in ms_ASC^PYD^ #2, #3 and #4) are located at the center of this pocket and make multiple van der Waals contacts with the side chains of Arg5_t_, Leu9_t_ and Leu50_t_ (Fig. S2), thus anchoring the peptide to the ASC^PYD^ surface. A more favorable binding energy for all the stapled peptides designed from the ASC^PYD^ template compared to that of the ASC^PYD^:ASC^PYD^ association (Table 1) suggested that they should successfully compete with ASC^PYD^ or even NLRP3^PYD^ monomers to bind the target ASC^PYD^ type-Ia interface.

### ASC peptides suppress production of IL-1β from human monocytic cells

**Figure 6:**
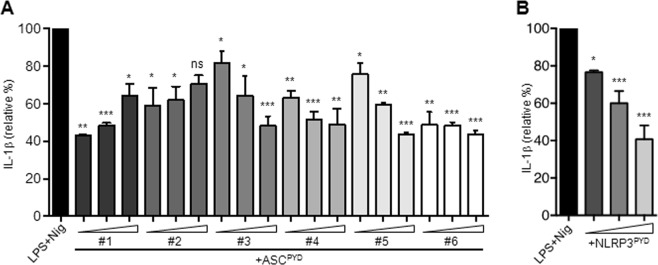
ASC peptides inhibit the release of IL-1β in THP-1 cells. Production of IL-1β from THP-1 cells pre-treated with ASC peptides ASC^PYD^ #1–6 (**A**) or NLRP3^PYD^ (**B**) (10, 50 and 100 μM) and stimulated with LPS and nigericin as measured by ELISA. Data are expressed as the means ± standard error relative to the LPS and nigericin condition and are representative of three independent experiments. ^***^p < 0.001. Abbreviations: LPS, lipopolysaccharide; Nig, nigericin.

We next examined whether the ASC peptides could inhibit IL-1β production, which is regulated by the inflammasome. We used THP-1 human monocytic cells to study inflammasome activation as they express high levels of NLRP3, ASC, and pro-casp-1. THP-1 cells were incubated with ms_ASC^PYD^ #1–6 or ms_NLRP3^PYD^ peptides at increasing concentrations for 4 hours and were then stimulated with LPS for 4 hours, followed by the NLRP3 activator nigericin for 1 hour. IL-1β levels were measured in the culture supernatant. LPS/nigericin treatment induced a robust IL-1β release (Fig. 6A). The ms_ASC^PYD^ peptides reduced IL-1β production from THP-1 cells in response to LPS/nigericin stimulation to variable degrees (Fig. 6A). Similarly, THP-1 cells pre-incubated with the ms_NLRP3^PYD^ peptide produced less IL-1β in a dose-dependent manner (Fig. 6B), indicating that NLRP3 peptides also can efficiently inhibit IL-1β release from human monocytic cells. We decided to continue investigating in detail the inhibitory effects of ms_ASC^PYD^ #5 and #6 peptides, as their estimated binding energies matched well with the experimental data of suppression of ASC-dependent IL-1 release in a dose-dependent manner. Since ms_ASCPYD #6 is the shorter version of ms_ASCPYD #5, this selection also enabled us to verify the effect of varying peptide lengths.

We note a crucial difference between ms_ASC^PYD^ #2 and ms_ASC^PYD^ #5 (or ms_ASC^PYD^ #6) is the presence of a negatively charged Glu at position 4 which would restrict the positioning of the crucial Arg (position 3) to interact with the protein. Indeed, docking simulations for ms_ASC^PYD^ #2 predicted a lower number of native-like conformations compared to ms_ASC^PYD^ #5 (28% vs. 38%, Fig. S3), whereas residue-wise binding energy analysis predicted unfavorable (positive) energy contribution for that Glu (Fig. S3) indicating that the negative charge is responsible to impede the binding of ms_ASC^PYD^ #2. Although ms_ASC^PYD^ #2 was not designed as a negative control, it serves as such due to its inability to suppress IL-1β release.

### ASC peptides effectively penetrate human monocytic cells

**Figure 7:**
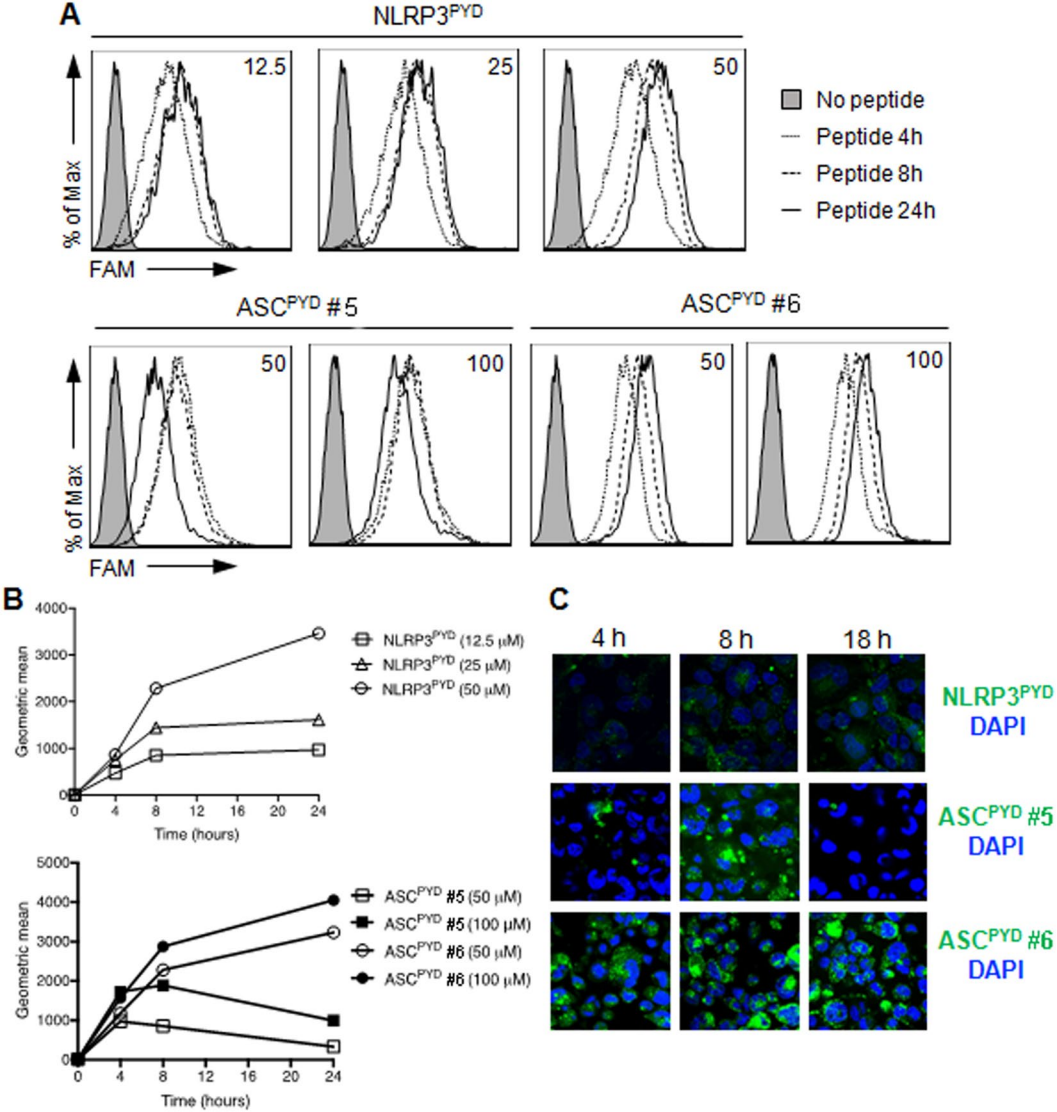
Internalization of ASC peptides. (**A**,**B**) THP-1 cells were incubated with FAM-NLRP3^PYD^ (12.5, 25 and 50 μM) (**A**), FAM-ASC^PYD^ #5 or FAM-ASC^PYD^ #6 (50 and 100 μM) (**B**) peptides at 37 °C for 4, 8 or 24 h. The cells were harvested, washed twice with PBS and then subjected to flow cytometry. Results of three independent experiments are shown as representative histograms of fluorescence intensity (A) and graphs showing the geometric mean intensity (**B**). (**C**) Imaging of THP-1 cells incubated with FAM-NLRP3^PYD^ (100 μM), FAM-ASC^PYD^ #5 or FAM-ASC^PYD^ #6 (50 μM) peptides at 37 °C for 4, 8 and 18 h. Nuclei were stained with DAPI. At least four different images were taken of each condition at an original magnification of objective 40× . Results are representative of three independent experiments.

As NLRP3 and ASC are cytosolic proteins, we examined whether the designed peptides could be taken up by monocytic cells to exert their biological effects. To assess peptide internalization, ms_NLRP3^PYD^, ms_ASC^PYD^ #5 and #6 peptides were labeled with the FAM fluorophore at the N-terminus. Internalization of the FAM-labeled peptides was measured as the mean fluorescence intensity (MFI) by fluorescence-assisted cell sorting (FACS). THP-1 cells were incubated with FAM-labeled ms_NLRP3^PYD^ (12.5, 25 and 50 μM), ms_ASC^PYD^ #5 and #6 peptides (50 and 100 μM) for 4, 8 and 24 hours. The internalization of the three FAM-labelled peptides was concentration dependent (Fig. 7A,B). When THP-1 cells were incubated with FAM-ms_NLRP3^PYD^ and FAM-ms_ASC^PYD^ #6 peptides at 37 °C, the fluorescent signal increased over time and reached its maximum intensity at 24 hours (Fig. 7A,B). Although the ms_ASC^PYD^ #5 peptide penetrated THP-1 cells to a similar extent at 4 hours, the fluorescent intensity subsequently decreased over-time (Fig. 7B). We also examined the internalization of FAM-labeled peptides in THP-1 cells by confocal microscopy. THP-1 cells were incubated with FAM-ms_NLRP3^PYD^ (100 μM), FAM-ms_ASC^PYD^ #5 or FAM-ms_ASC^PYD^ #6 (100 μM) for 4, 8 and 18 hours. FAM-labeled peptides localized primarily in the cytosol, but not in the nucleus, of THP-1 cells (Fig. 7C). The ms_NLRP3^PYD^ and ms_ASC^PYD^ #5 remained stable for up to 18 hours, while ms_ASC^PYD^ #6 was degraded and slowly disappeared (Fig. 7C). These results indicate that the ASC peptides can be effectively internalized by human monocytic cells, but that their intracellular stability can vary depending on the peptide sequence.

### ASC peptides prevent IL-1β and IL-18 production by blocking ASC oligomerization

**Figure 8:**
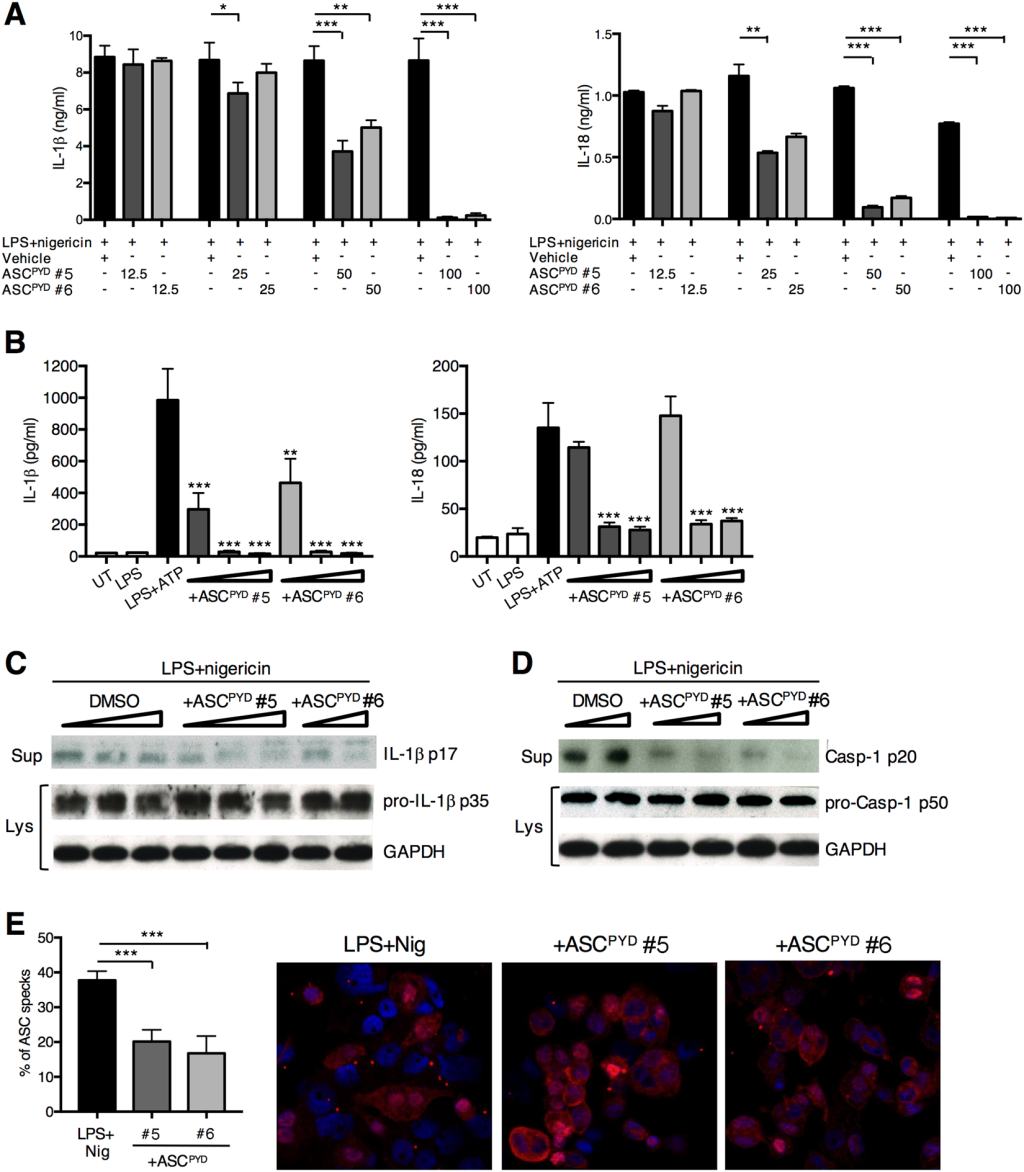
ASC peptides effectively block IL-1β and IL-18 release and ASC oligomerization in human blood monocytes. (**A**) IL-1β and IL-18 were measured in cell-free supernatants from THP-1 cells incubated with FAM-ASC^PYD^ #5 or FAM-ASC^PYD^ #6 (12.5, 25,50 and 100 μM) for 24 h and primed with LPS (0.1 μg/ml, 4 h) followed by stimulation with nigericin (10 μM, 1 h), as assessed by ELISA. (**B**) Production of IL-1β and IL-18 from human blood monocytes stimulated or not with LPS (0.1 μg/ml, 4 h) alone or in a combination of ATP (5 mM, 30 min) in the presence of FAM-ASC^PYD^ #5 or FAM-ASC^PYD^ #6 (25, 50 and 100 μM) peptides. (**C**,**D**) Western blot analysis of processed IL-1β p17 (**C**) and Casp-1 p20 (**D**) in cell-free supernatants of THP-1 cells primed with LPS (0.1 μg/ml, 4 h), followed by stimulation with nigericin (10 μM, 1 h) in the presence of FAM-ASC^PYD^ #5 (25, 50 and 100 μM), FAM-ASC^PYD^ #6 (50 and 100 μM) peptides, or DMSO vehicle. Expression of pro-IL-1β p35 and pro-Casp-1 p50 was assessed in cell lysates. GAPDH was used as loading control. (**E**) Imaging of ASC speck oligomerization in THP-1 cells treated with LPS (0.1 μg/ml, 4 h) and nigericin (10 μM, 1 h) in the absence or presence of FAM-ASC^PYD^ #5 or FAM-ASC^PYD^ #6 (100 μM) peptides. The number of ASC specks were counted manually in at least 100 cells, and the proportion of cells containing a speck was shown. Objective 100× , Magnification 1.0× . Data represent the mean ± standard error of triplicate wells and are representative of three independent experiments. *p < 0.05; **p < 0.01; ***p < 0.001.

Incubation of THP-1 cells with ms_ASC^PYD^ #5 or ms_ASC^PYD^ #6 resulted in a dose-dependent suppression of IL-1β production, resulting in >60% and >90% inhibition at peptide concentrations of 50 and 100 μM, respectively (Fig. 8A). We also analyzed the production of IL-18 — another cytokine that requires inflammasome-mediated activation to exert its biological activity. Both ms_ASC^PYD^ #5 and ms_ASC^PYD^ #6 peptides decreased IL-18 production by LPS/nigericin-stimulated THP-1 cells in a dose-dependent manner (Fig. 8A). Similar to THP-1 cells, we found that treatment of LPS-primed primary human monocytes with ms_ASC^PYD^ #5 and ms_ASC^PYD^ #6 peptides effectively inhibited both IL-1β and IL-18 secretion upon ATP stimulation (Fig. 8B), suggesting that ASC peptides may inhibit NLRP3 inflammasome activation.

To examine whether ASC peptides interfere with NLRP3 inflammasome activation, we first assessed processing of pro-IL-1β. The amount of processed IL-1β was inhibited in supernatants from ms_ASC^PYD^ #5 and ms_ASC^PYD^ #6 peptide treated THP-1 cells, in a dose-dependent manner (Fig. 8C). Similarly, treatment with ms_ASC^PYD^ #5 and ms_ASC^PYD^ #6 peptides reduced caspase-1 activation in a dose-dependent manner, evaluated as caspase-1 p20 in supernatants from THP-1 cells stimulated with LPS/nigericin (Fig. 8D). These data support that ASC peptides inhibit caspase-1 activation by NLRP3. The ASC peptides did not consistently affect pro-IL-1β or pro-caspase-1 expression in cell lysates (Fig. 8C,D), indicating that ASC peptides did not interfere with the priming step of NLRP3 inflammasome activation.

We next examined the effect of ASC peptides on NLRP3-dependent ASC oligomerization — a pivotal event in NLRP3 inflammasome activation. Upon NLRP3 activation by LPS/nigericin, ASC condensed into a large speck in each cell, but pre-treatment with ms_ASC^PYD^ #5 and ms_ASC^PYD^ #6 significantly inhibited speck formation (Fig. 8E). Collectively, these results indicate that the ASC peptides designed in our study efficiently inhibit NLRP3 inflammasome activation by interfering with NLRP3-induced ASC oligomerization, thereby reducing caspase-1 activation and IL-1β and IL-18 production from human monocytes/macrophages.

## Discussion

Many inflammasome receptors require the adaptor protein ASC to form oligomeric filaments, which is an essential intermediate step to activate caspase-1. We, therefore, proposed that ASC may be a suitable target for ASC-dependent inflammatory pathways to disrupt excessive IL-1β production. From a structural view, spatial assemblies and monomer subunits of ASC^PYD^ in the filament structure are conserved between human and mouse, implying the functional importance of its polymerization mechanism. The ASC^PYD^ monomer is an all-α protein composed of six helices, suggesting that helical peptides would be potent binders. The flat and charged binding surface of ASC^PYD^ also renders helical peptides more suitable to bind compared to small molecule-based inhibitors. Structural and mutational studies showed that electrostatic interactions are a dominant driving force for ASC^PYD^ filament formation^32,34,38^. The ASC mature filaments are stabilized by three types of interactions between the PYD subunits, with the type I (H1–H4 helices of one PYD binding to H2–H3 of the other PYD) as dominant and found both in the cryo-electron microscopy structures^32,34^ and NMR analysis^39^, specifying the reactivity of the type I surface. Utilizing a charge complementation approach, we designed peptides composed of positively charged residues to target the negatively charged type I surface of ASC^PYD^. We introduced a larger hydrophobic side chain (Leu to Ile/Phe) to increase the peptide affinity for the flat surface; this modification optimized packing inside the hydrophobic pocket on the ASC^PYD^ surface, thus anchoring the peptides. This small hydrophobic pocket had an important role in the interaction with the peptides. This agrees with previous findings where the size and shape of the ASC filament were altered by the L25A mutation and recovered back by the L25M mutation^38^, suggesting a key function of these hydrophobic interactions at the core of the type-I interface in ASC filament formation. Major issues dealing with linear helical peptides include loss of their helical conformation in solution, faster proteolytic degradation, and reduced cell permeability. Stapling at the I,I + 7 positions ensures that the unbound peptide retains its helical conformation in solution thus minimizing the loss in conformational entropy upon binding. The presence of positively charged residues is advantageous when considering cell membrane permeability of the peptides^40^; stapling itself is also helpful in increasing cellular uptake of peptides^40^.

We designed and experimentally validated a set of stapled peptide inhibitors targeting the PYD domain of ASC with the aim to block inflammasome formation and inhibit IL-1β and IL-18 release. These peptides were based on the ASC^PYD^ helix 2 sequence and were developed to competitively target the protein-protein interaction between ASC^PYD^ molecules. Specific structural factors such as charge distribution, the location of the hydrophobic patch and the different lengths of certain α-helices put together make the type I surface more reactive which should facilitate these positively charged stapled peptides to specifically bind to the negatively charged type I surface. As such, measuring peptide binding to ASC^PYD^ by binding assays is difficult due to the highly aggregating features of ASC^39,41^. Starting from the ASC^PYD^-peptide structural model built from the ASC^PYD^ filament structure, we confirmed by coarse-grained docking simulations that the designed peptides could bind to the target binding site and did not bind to other similar death domains such as ASC^CARD^. The complex structure was analyzed by atomistic MD simulation to guide the design of a set of stapled peptides with improved binding affinities. In contrast to the unstapled wild-type counterparts, the stapled peptides retained their helical conformations in solution. Notably, the prior organization of the peptides into conformations similar to the ones they adopt upon binding to their targets results in lowering the entropic costs associated with embedding the peptide free in solution onto a surface. Tested stapled peptides were cell-permeable, and were able to suppress the IL-1β and IL-18 production by human monocytic cells (THP-1 cells and blood monocytes). We discovered that ASC peptides blocked NLRP3 inflammasome activation by preventing ASC oligomerization, which in turn suppressed casp-1 activation and thereby IL-1β release. The stapled peptides did not interfere with the priming (signal 1) of the NLRP3 inflammasome, as pro-IL-1β expression remained unaffected. Peptides did not induce significant cell death compared to THP-1 cells treated by the vehicle control treated cells (data not shown).

Our proof-of-concept study is the first to use stapled peptides to block the NLRP3 inflammasome. Peptide inhibitors designed to mimic known sequences of caspase substrates effectively suppress caspase-dependent effects in various cells. In particular, caspase-1 is activated in different inflammasome complexes and several short peptide inhibitors have been developed^42^. However, due to the high similarity between caspase sequences, many peptide inhibitors designed to target caspase-1 also block the activity of other caspases, making data interpretation difficult. For example, the cell-permeable and irreversible caspase-1 inhibitor Y-VAD also inhibits caspase-4 and caspase-5. A small number of reversible specific inhibitors of caspase-1 (including VX-740 and VX-765) have entered clinical trials^42,43^: preclinical studies demonstrated that these inhibitors ameliorate disease severity in models of arthritis and inflammation, but some were withdrawn from clinical trials because of liver toxicity after long-term administration in animal studies. As such, no caspase inhibitors have yet reached Phase III.

Numerous research efforts have aimed to identify small molecules that can prevent inflammasome activation, including those that can inhibit the NLRP3 inflammasome^18,20,21,24^. However, the molecules identified to date are nonspecific and thus may interfere with several mechanisms that indirectly inhibit inflammasome activation. Peptide therapeutics is a growing area of interest as attractive alternatives to small-molecule inhibitors mainly due to their high potency and specificity^44^. Since the template sequence of the designed peptides is taken from the target molecule itself, in principle, they are less likely to initiate an immune response. We note that the effective stapled peptide dose used here (50–100 μM) are comparable to the average *K*_*D*_ value for ASC^PYD^ self-association (65 μM) measured by NMR titration^39^.

This work provides the first example of using peptide-based modulators to target components of the inflammasome pathway. The ability of the constrained helical peptides to inhibit the PYD interaction provides a good starting point to design similar peptide-based inhibitors for the other death domain families. The promising aspect will be addressed in future investigations aimed at understanding whether the ASC peptides designed in our study can block the activity of other PYD proteins. Studies addressing the bioavailability of these peptides would throw light on their potential as therapeutics.

## Methods

### Study approval

Ethical approval for all blood sources and processes used in this study was obtained from the National University of Singapore Institutional Review Board (license: NUS-IRB 12-044E). Subjects gave written informed consent in accordance with the Declaration of Helsinki. All experiments were carried out following the approved guidelines and regulations.

### Structure modeling

The ASC^PYD^ -ASC^PYD^ dimer (interacting through the type-I interface) obtained from the human ASC^PYD^ filament structure solved by cryo-electron microscopy at atomic resolution (3J63.pdb, 3.8 Å) was used for the structural modeling. There the conformation of helix 2 peptide (^16^TAEELKKFKLKLLSV^30^) of the type-Ib monomer bound to the type-Ia interface of the other monomer was used as the initial model structure to design the stapled peptides (Fig. 1A). A hydrocarbon staple across positions 5 and 12 (I, I + 7) along the peptide was constructed. The peptide was mutated in silico at various positions (Table 1) and the resulting models subject to detailed optimization. This included carrying out extensive (3 independent simulations of 200 ns each per protein-peptide system) molecular dynamics simulations on the complexes in the aqueous phase followed by detailed structural and energetic analysis (described below).

### Docking simulations

All the designed peptides have positive electrostatic potential due to the presence of four/five positively charged residues. Conversely, the ASC^PYD^ type-Ia interface is negatively charged. We used coarse-grained simulations (GO model type) to explore the binding of the designed peptide ms_ASC^PYD^ #5 to ASC^PYD^, where the interaction between the protein and the peptide was modeled solely by electrostatic interactions between charged residues. Here coarse-grained simulations are advantageous over all-atom MD simulations in enabling access to timescales long enough to achieve the sampling of the binding process. Overall, we followed an approach to protein modeling similar to that described in previous studies^45,46^. Each protein residue was represented by two beads placed at the Cα- and Cβ-positions. Charged residues (K, R, H, D, and E) had a charge at the Cβ-position. The native topology of the protein and peptide was retained by applying a Lennard–Jones (L-J) potential during the simulation. The electrostatic interaction between charged residues was modeled by applying Debye–Hückel potential. As ASC^PYD^ does not undergo significant conformational changes when interacting, all simulations were run at relatively low temperatures to allow the protein and peptide to fluctuate around their native states. The salt concentration was also kept low (10 mM) to reduce the charge screening effect. Since the distances between the charged beads in the coarse-grained model are longer than the corresponding distances in the all atom representations, the effective salt concentration is higher here, and 10 mM is equivalent to a salt concentration of ~30 mM. Each predictive simulation was started by placing the unbound peptide at one of 10 different positions at about ~35 Å (distance between the protein surface and the peptide center), ensuring that the peptides were distributed well around the protein for unbiased sampling. Each trajectory was simulated for 10^7^ steps, and a total of 100 simulations (10 simulations for each of the 10 initial positions of the peptide around the protein) were run for adequate sampling. Coordinates were saved every 1000 steps and only the last 2000 conformations were collected during each simulation, resulting in a total of 2 × 10^5^ analyzed conformations. As the number and positions of the positive residues are similar in all the peptides, all other peptides are expected to exhibit a similar binding behavior in docking simulations.

The simulated conformations were compared with the experimental type-I ASC^PYD^ interface structure (Fig. 1A) to evaluate their binding accuracy. A structural similarity parameter (D) was calculated to quantify the degree of deviation of the simulated conformation from the experimental structure. We used a similar approach as described in a previous study^47^. Only Cβ beads of the interface residues of the protein (Asp6, Leu9, Asp10, Glu13, Asp48, Leu50, Asp51, and Asp54) and peptide (Lys6, Lys7, Lys9, Ile10, and Lys11) were considered for calculating D using the following equation:

$$D=\frac{1}{{N}_{protein}{N}_{peptide}}{\sum }_{i}^{{N}_{protein}}({\sum }_{j}^{peptide}{r}_{ij}-{\sum }_{j}^{peptide}{r}_{ij}^{0})$$

where, i and j stand for the i^th^ Cβ interface bead of the protein and the j^th^ Cβ interface bead of the peptide, respectively, and *r*_*ij*_ and ${r}_{ij}^{0}$ are the distances between those bead pairs in the simulated and the experimental structure, respectively. *N*_*protein*_ and *N*_*peptide*_ are the number of protein and peptide interface residue beads, respectively. D quantifies the overall conformational similarity between the predicted binding interface of the peptide:ASC^PYD^ complex and the experimental type-I interface structure (only helix 2 from the positive surface). The direction of the peptide with respect to the ASC^PYD^ interface is considered when calculating pair-wise distances. Finally, to obtain a detailed structural quantification, interface residues were divided into two spatial groups that cover the two halves of the peptide, and D was calculated for each (termed as D_1_ and D_2_). A low D_1_ or D_2_ value indicates high conformational similarity.

The same simulation and sampling strategies as outlined above were used to explore the interactions between ASC^CARD^ (2KN6.pdb, residue:115–195) and ms_ASC^PYD^ #5. The distances found in the simulated conformations suggests that the peptide does not bind to ASC^CARD^.

### Molecular dynamics (MD) simulations

The bound conformation of helix 2 (^16^TAEELKKFKLKLLSV^30^) to ASC^PYD^ (Fig. 1A) was used as the initial model structure for all-atom MD simulations of peptide-protein complexes (Table 1). The ASC^PYD^ type-I protein-protein complex structure was prepared by taking subunit A and B from the filament (3J63.pdb) for simulation. For different peptides, residues at different positions were mutated (Table 1) in the starting structure while keeping the binding posture of the peptide unaltered. The hydrocarbon linker (staple) in the various designed stapled peptides ((2R)-2(7-octenyl) alanine and (2S)-2(4-pentenyl) alanine) was modeled using the XLEAP module of AmberTools12. The RESP (Restrained Electrostatic Potential) based atomic charges of the hydrocarbon linker were derived from the R.E.D. server using the RESP-A1A (HF/6–31G*) charge model and Guassian_2009_C.01 quantum mechanics program^48^. Other force field parameters were derived from all-atom ff99SB^49^ force field in Amber12^50^. The N-terminus of the protein and peptide was acetylated, the C-terminus of the protein was methylated, and the C-terminus of the peptide was amidated. The starting structures were placed in a cuboid water box such that the minimum distance from the edge of the box was at least 10 Å. The TIP3P water model was used for solvation^51^. The solvated systems were neutralized by adding chloride ions using the LEaP module of AmberTools12. A total of 10 peptide-protein complex systems (seven stapled and three unstapled) and one protein-protein complex system were prepared (Table 1), all of which were then used in the subsequent MD simulation steps.

Energy minimizations and MD simulations were carried out using the PMEMD module of AMBER12 employing the all-atom ff99SB force field parameters. Three independent explicit-solvent MD simulations using different initial atomic velocities were carried out for each of the eleven systems to improve the sampling. The solvated systems were initially relieved of any unfavorable interactions by subjecting them to 1,000 steps of energy minimization, which involved using 500 steps each of steepest descent, followed by conjugate gradient algorithms. The conjugate gradient algorithms were used as follows: i) first, Cartesian restraints were imposed on the solute while the solvent molecules were then allowed to relax around it, ii) next, the solvent was restrained and the solute was energy minimized, iii) finally, no restraints were imposed and the whole system was minimized. A force constant of 500 kcal mol^-1^ Å^2^ was used during the restraining steps. The systems were then gradually heated to 300 K over a period of 30 ps using the NVT ensemble. Following this, each system was equilibrated under NPT conditions for 500 ps. They were then subjected to the production phase of MD simulation using the NPT ensemble for a period of 200 ns each. This procedure resulted in 33 (11 × 3) simulations with a combined simulation period of 6.6 μs. The simulation temperature of 300 K was set using Langevin dynamics^52^, with a collision frequency of 2.0 ps^−1^. The pressure was maintained at 1 atm using weak-coupling^53^ with a pressure relaxation time of 2 ps. Periodic boundary conditions in x, y and z directions were appropriately applied. The particle mesh Ewald (PME) method was used to treat electrostatic interactions^54^ with a grid spacing of 1.0 Å. The SHAKE algorithm^55^ was applied to constrain all bonds involving hydrogen atoms, allowing for a time step of 2 fs. A cutoff distance of 9 Å was implemented for nonbonded interactions. We note that all the peptides remained in bound conditions similar to the starting conformation throughout the simulations. Coordinates were saved every 10 ps.

### Binding energy decomposition and salt-bridge distance analysis

The contribution of each peptide residue to the binding energy of complex formation was calculated by applying the free energy decomposition method^56^ on 2,000 equally-spaced structures extracted from the MD simulation trajectories. Binding energies were computed by the MMPBSA.py script^57^ in AMBERTools12 using the MM/GBSA (Molecular Mechanics/Generalized Born Surface Area) method. Water molecules and Cl^−1^ ions were stripped from the extracted structures, and the solvent effect was represented using a Generalized Born Solvation Model^58^. A salt concentration of 150 mM was used. The nonpolar contribution to solvation free energy was estimated from the solvent accessible surface area (SASA) using the ICOSA method. Energy values are represented in Table 1 as an average of the values obtained from each of the three simulation runs. Distance calculations from the trajectories were performed using the PTRAJ module in AmberTools12.

### Peptide synthesis

The linear and stapled peptides were synthesized by American Peptide Company, Inc. (Sunnyvale, CA USA) by replacing the two respective Leu residues at positions I and I + 7 of the linear peptide with the olefin-bearing unnatural amino acids (R)-2-(7′octenyl) alanine and (S)-2-(4′-pentenyl) alanine, respectively, and stapled via olefin metathesis using the Grubbs catalyst. All peptides were purified using HPLC to >90% purity. They were amidated at their C-terminus and acetylated at their N-terminus. Peptides with FAM-labeled at the N-terminus were also synthesized by American Peptide Company, Inc. with the C-terminal amidated and was purified using HPLC to >90% purity.

### Cell stimulation

Human monocytic THP-1 cells (1 × 10^7^ cells) were plated in a 10-cm tissue culture dish and treated with PMA (200 nM) overnight to differentiate monocytic cells into macrophages. Cells were then washed and allowed to rest in fresh media for another day. For stimulation, differentiated THP-1 cells were trypsinized and seeded in 96-well plates (2 × 10^6^/well). Peripheral blood mononuclear cells were isolated by Ficoll-Hypaque density gradient centrifugation from buffy coats obtained from anonymous donors provided by the National University Hospital Blood Bank, Singapore. Isolation of monocytes was performed using negative selection with the Monocyte Isolation Kit II (Miltenyi Biotech), following the manufacturer’s instructions. Monocytes were plated in tissue culture non-treated 96-well plates (2.5 × 10^5^/well) in 250 µl RPMI 1640-Glutamax media supplemented with 3% human AB serum (Gemini Bio Products).

THP-1 cells and monocytes were incubated with NLRP3^PYD^ (12.5–25–50 μM), ASC^PYD^ #5 (12.5-25-50-100 μM) or ASC^PYD^ #6 (12.5-25-50-100 μM) for the indicated durations. Cells were then washed once with PBS and primed with *E*. *coli* LPS (0.1 μg/ml, Invivogen) for 4 h followed by stimulation with nigericin (10 μM) for an additional 1 h or ATP (5 μM) for an additional 30 min. The cell culture supernatants were then collected and centrifuged at 500 × *g* to remove cells and cellular debris.

### ELISA

IL-1β levels were measured using antibodies from eBioscience (cat. #14-7018-81 and #13-7016-85), following the manufacturer’s instructions. IL-18 levels were measured using antibodies from MBL (D044-3 and D045-6) following the manufacturer’s instructions. The absorbance was measured at 450 nm with the reference wavelength at 570 nm, using an M200 Infinite plate reader (Tecan).

### Flow cytometry

THP-1 cells (0.5 × 10^6^) were incubated with FAM-NLRP3^PYD^ (12.5-25-50 μM), FAM-ASC^PYD^ #5 or FAM-ASC^PYD^ #6 (50-100 μM) peptides in a 6 well-plate. After 4, 8 and 24 h incubation, THP-1 cells were washed twice with PBS and harvested. The analysis was performed on a BD FACSCANTO II (BD Biosciences), and the data were analyzed using FlowJo (Treestar).

### Confocal microscopy

THP-1 cells (0.4 × 10^6^ cells) were seeded into chambers of an 8-well IBIDI µ-Slide. Cells were incubated with FAM-NLRP3^PYD^ (12.5-25-50 μM), FAM-ASC^PYD^ #5 or FAM-ASC^PYD^ #6 (50–100 μM) peptides for 4, 8 and 18 h. The cells were then washed twice with PBS and fixed with 4% PFA for 10 min before permeabilization and incubation with an ASC antibody (4 µg/ml, Santa Cruz Biotechnology, 22514-R) in blocking/permeabilization buffer (10% goat serum, 1% FBS and 0.5% Triton-X in PBS) for 1 h. The cells were subsequently washed three times with blocking/permeabilization buffer prior to incubation with Alexa Fluor 568-labeled goat anti-rabbit antibody (1 µg/ml, Invitrogen, A11011) together with DAPI for 30 min, and imaged on an FV1000 Olympus confocal microscope using an x100 objective with an additional zoom of 2. Z-stack images were acquired and the maximum projections are shown.

### Western blot

Cell-free supernatants were mixed 1:4 to cold acetone and incubated at −20 °C for at least 1 h. The mixtures were then centrifuged at 15,000 rpm for 15 min at 4 °C. Supernatants were discarded and pellets re-suspended in 1X Laemmli buffer. Total cell lysates were prepared using RIPA buffer containing 150 mM NaCl, 0.5% sodium deoxycholate, 0.1% SDS, 50 mM Tris-HCl pH 8.0 and 1% Triton-X-100. Protein concentrations were determined by BCA assay (Thermo Scientific, cat. 23227) following the manufacturer’s instructions. Lysates were reconstituted in Laemmli buffer (20–50 μg), boiled and separated by SDS–PAGE, and transferred onto PVDF membranes. Membranes were blocked with blocking buffer consisting of 5% non-fat dry milk in phosphate-buffered saline (PBS) with 0.1% Tween 20 (PBST) for 1 h at room temperature, and incubated overnight at 4 °C with anti-caspase-1 antibody (1:500; Cell Signaling, D7F10), anti-IL-1β antibody (1:1000; Santa Cruz, sc-7884) and anti-GAPDH antibody (1:2000; Millipore, #MAB374) in blocking buffer. Blots were then washed six times with PBST (PBS with 0.1% Tween-20). Secondary antibodies were as follows: goat anti-rabbit-HRP (1:5,000; Santa Cruz, sc-2004) and goat anti-mouse-HRP (1:5,000; Millipore, AP124P).

### Statistical analysis

Statistical significance was assessed by unpaired two-tailed t-tests or one-way ANOVA. Data were analyzed using Prism 7 (GraphPad).

## Supplementary information

Inhibition of NLRP3 inflammasome activation by cell-permeable stapled peptides

## Data Availability

All the data are available upon request.
