# Supplementary material for: Inhibition of NLRP3 inflammasome activation by cell-permeable stapled peptides
Source: Sci Rep. 2019 Mar 20;9:4913. doi: 10.1038/s41598-019-41211-3 (PMC6426897; doi:10.1038/s41598-019-41211-3)

**SUPPLEMANTARY INFORMATION**

**Inhibition of the NLRP3 inflammasome activation by cell-permeable stapled peptides**

Arumay Pal^1,#^, Kurt Neo^2,#^, Lakshminarayanan Rajamani^3^, Fernando Jose Ferrer^4^, David P Lane^4^, Chandra Verma^1,5,6,*^ and Alessandra Mortellaro^2,7,*^

^1^ Bioinformatics Institute (BII), Agency for Science, Technology and Research (A*STAR), 30 Biopolis Street, #07-01 Matrix, Singapore 138671.

^2^ Singapore Immunology Network (SIgN), Agency for Science, Technology and Research (A*STAR), 8a Biomedical Grove, Singapore 138648.

^3^ Singapore Eye Research Institute (SERI), The Academia, 20 College Road, Discovery Tower Level 6, Singapore 169856.

^4^ p53Lab, Agency for Science, Technology and Research (A*STAR), 8A Biomedical Grove, #06-04/05 Neuros/Immunos, Singapore 138648.

^5^ School of Biological Sciences, Nanyang Technological University, 60 Nanyang Drive, Singapore 637551.

^6^ Department of Biological Sciences, National University of Singapore, 16 Science Drive 4, Singapore 117558.

^7^ San Raffaele Telethon Institute for Gene Therapy (SR-Tiget), IRCCS San Raffaele Scientific Institute.

^#^ These authors contributed equally to this work

* These authors share senior authorship

Corresponding authors: Alessandra Mortellaro, San Raffaele Telethon Institute for Gene Therapy (SR-Tiget), IRCCS San Raffaele Scientific Institute, Via Olgettina 58, 20132 Milan, Italy. mortellaro.alessandra@hsr.it; Chandra Verma, Bioinformatics Institute (BII), Agency for Science, Technology and Research (A*STAR), 30 Biopolis Street, Matrix #07-01, Singapore 138671, chandra@bii.a-star.edu.sg

**SUPPLEMENTARY TABLES**

**Table S1.** Analysis of the residue-wise binding energy decomposition for the ASC^PYD^ - ASC^PYD^ type-I interface, calculated from MD simulations of their complex. Residues making significant contributions to the total binding energies are shown.

| ASC^PYD^ - ASC^PYD^ type-I interface | | | |
| --- | --- | --- | --- |
| Interface Type Ia  (-ve surface) | | Interface Type Ib  (+ve surface) | |
| Residue | ΔGcalc (kcal/mol) | Residue | ΔGcalc (kcal/mol) |
| Met1 | -4.71 ± 1.91 | Lys21 | -1.80 ± 1.34 |
| Arg3 | -3.09 ± 2.76 | Lys22 | -2.81 ± 1.21 |
| Arg5 | -1.96 ± 0.85 | Leu25 | -4.25 ± 0.62 |
| Asp6 | -1.39 ± 1.08 | Lys26 | -3.55 ± 1.19 |
| Leu9 | -1.54 ± 0.49 | Leu28 | -1.24 ± 0.48 |
| Leu50 | -3.27 ± 0.65 | Ser29 | -2.83 ± 1.47 |
| Asp51 | -1.06 ± 0.85 | Val30 | -1.16 ± 0.84 |
|  |  | Arg41 | -0.98 ± 0.71 |

**Table S2.** Analysis of the residue-wise binding energy decomposition for the designed peptides, calculated from MD simulations of their complexes with ASC^PYD^. For the protein, only the residues making significant contributions to the total binding energies are reported.

| ms_ASC^PYD^ #1 | | | | ms_ASC^PYD^ #2 | | | |
| --- | --- | --- | --- | --- | --- | --- | --- |
| Peptide | | Protein | | Peptide | | Protein | |
| Residue | ΔGcalc (kcal/mol) | Residue | ΔGcalc (kcal/mol) | Residue | ΔGcalc (kcal/mol) | Residue | ΔGcalc (kcal/mol) |
| Thr1 | -0.36 ± 0.95 | Met1 | -2.90 ± 0.92 | Thr1 | 0.08 ± 0.03 | Met1 | -0.40 ± 1.02 |
| Ala2 | 0.09 ± 0.42 | Gly2 | -2.38 ± 0.90 | Ala2 | 0.08 ± 0.03 | Arg 3 | -1.53 ± 1.26 |
| Glu3 | 0.59 ± 0.48 | Arg 3 | -0.76 ± 0.58 | **Arg3** | **-10.18 ± 2.34** | Arg5 | -3.52 ± 1.88 |
| Glu4 | 0.62 ± 0.33 | Arg5 | - 1.13 ± 0.73 | Glu4 | 0.60 ± 0.24 | Asp6 | -1.45 ± 1.21 |
| Lys6 | -0.68 ± 1.68 | Asp6 | -1.24 ± 1.33 | Lys6 | -0.09 ± 0.71 | Leu9 | -1.90 ± 0.46 |
| **Lys7** | **-2.73 ± 1.61** | Leu9 | -2.11 ± 0.79 | **Lys7** | **-2.36 ± 1.01** | Glu13 | -5.46 ± 1.89 |
| Phe8 | -0.16 ± 0.09 | Glu13 | - 0.87 ± 1.02 | Ala8 | -0.11 ± 0.05 | Asp48 | -3.40 ± 1.07 |
| Lys9 | 0.21 ± 0.30 | Leu50 | - 2.61 ± 1.03 | Lys9 | 0.11 ± 0.16 | Leu50 | -4.38 ± 0.58 |
| **Leu10** | **-4.49 ± 0.86** |  |  | **Phe10** | **-6.09 ± 1.17** |  |  |
| **Lys11** | **-2.48 ± 1.00** |  |  | **Lys11** | **-2.13 ± 0.99** |  |  |
| **Leu13** | **-2.98 ± 0.99** |  |  | Glu13 | 0.09 ± 1.19 |  |  |
| **Ser14** | **-2.12 ± 1.02** |  |  | **Ser14** | **-2.83 ± 1.41** |  |  |
| **Val15** | **-2.66 ± 1.40** |  |  | **Val15** | **-1.55 ± 0.97** |  |  |
| ms_ASC^PYD^ #3 | | | | ms_ASC^PYD^ #4 | | | |
| Peptide | | Protein | | Peptide | | Protein | |
| Residue | ΔGcalc (kcal/mol) | Residue | ΔGcalc (kcal/mol) | Residue | ΔGcalc (kcal/mol) | Residue | ΔGcalc (kcal/mol) |
| Thr1 | 0.09 ± 0.06 | Met1 | -1.56 ± 1.31 | Thr1 | 0.10 ± 0.05 | Met1 | -1.60 ± 1.30 |
| Ala2 | 0.08 ± 0.04 | Gly2 | -1.05 ± 0.80 | Ala2 | 0.08 ± 0.03 | Gly2 | -0.65 ± 0.49 |
| **Arg3** | **-10.07 ± 2.01** | Arg5 | -2.70 ± 1.78 | **Arg3** | **-9.87 ± 1.94** | Arg5 | -2.82 ± 1.64 |
| Val4 | -0.07 ± 0.04 | Asp6 | -1.18 ± 1.10 | Ala4 | -0.06 ± 0.04 | Asp6 | -0.68 ± 1.30 |
| Lys6 | -0.13 ± 0.88 | Leu9 | -2.10 ± 0.53 | **Lys6** | **-0.70 ± 1.12** | Leu9 | -1.82 ± 0.52 |
| **Lys7** | **-2.09 ± 1.18** | Glu13 | -5.53 ± 1.78 | **Val7** | **-1.62 ± 0.52** | Glu13 | -3.06 ± 1.10 |
| Ala8 | -0.09 ± 0.04 | Asp48 | -3.46 ± 1.05 | Ala8 | -0.06 ± 0.05 | Asp48 | -3.40 ± 1.01 |
| Lys9 | 0.16 ± 0.36 | Leu50 | -4.16 ± 0.57 | Lys9 | 0.03 ± 0.32 | Leu50 | -3.82 ± 0.53 |
| **Phe10** | **-4.90 ± 1.00** |  |  | **Phe10** | **-5.87 ± 1.70** | Asp51 | -0.57 ± 0.75 |
| **Lys11** | **-2.08 ± 1.20** |  |  | **Lys11** | **-1.50 ± 1.17** |  |  |
| Glu13 | 0.06 ± 1.13 |  |  | Glu13 | -0.79 ± 0.83 |  |  |
| **Ser14** | **-1.16 ± 1.14** |  |  | **Ser14** | **-2.20 ± 1.66** |  |  |
| **Val15** | **-1.44 ± 1.14** |  |  | **Val15** | **-0.98 ± 0.75** |  |  |
| ms_ASC^PYD^ #5 | | | | ms_ASC^PYD^ #6 | | | |
| Peptide | | Protein | | Peptide | | Protein | |
| Residue | ΔGcalc (kcal/mol) | Residue | ΔGcalc (kcal/mol) | Residue | ΔGcalc (kcal/mol) | Residue | ΔGcalc (kcal/mol) |
| Thr1 | 0.08 ± 0.06 | Met1 | -1.14 ± 1.46 | **Arg1** | **-9.94 ± 2.07** | Met1 | -0.61 ± 1.13 |
| Ala2 | 0.09 ± 0.04 | Gly2 | -1.08 ± 0.88 | Ala2 | -0.04 ± 0.05 | Gly2 | -0.40 ± 0.91 |
| **Arg3** | **-10.15 ± 1.76** | Arg5 | -3.05 ± 1.93 | **Lys4** | **-0.91 ± 1.02** | Arg5 | -3.09 ± 1.80 |
| Ala4 | -0.06 ± 0.04 | Asp6 | -0.78 ± 0.99 | **Lys5** | **-2.03 ± 0.98** | Asp6 | -1.24 ± 1.15 |
| Lys6 | -0.80 ± 1.45 | Leu9 | -1.98 ± 0.46 | Ala6 | -0.12 ± 0.05 | Leu9 | -2.05 ± 0.48 |
| **Leu7** | **-2.06 ± 0.50** | Glu13 | -3.14 ± 1.14 | Lys7 | -0.17 ± 0.22 | Glu13 | -5.65 ± 1.60 |
| Ala8 | -0.07 ± 0.05 | Asp48 | -3.40 ± 1.03 | **Ile8** | **-5.43 ± 1.19** | Asp48 | -3.37 ± 1.22 |
| Lys9 | 0.13 ± 0.35 | Leu50 | -4.42 ± 0.59 | **Lys9** | **-2.29 ± 1.11** | Leu50 | -4.24 ± 0.55 |
| **Ile10** | **-5.50 ± 1.43** | Asp51 | -0.98 ± 1.03 | Glu11 | -0.25 ± 0.90 | Asp51 | -0.62 ± 0.73 |
| **Lys11** | **-1.20 ± 1.28** |  |  | **Ser12** | **-0.70 ± 0.98** |  |  |
| Glu13 | -0.07 ± 1.23 |  |  | **Val13** | **-1.59 ± 1.14** |  |  |
| **Ser14** | **-1.68 ± 1.47** |  |  |  |  |  |  |
| **Val15** | **-1.42 ± 1.22** |  |  |  |  |  |  |
| ms_NLRP3^PYD^ | | | |  | | | |
| Peptide | | Protein | |  | |  | |
| Residue | ΔGcalc (kcal/mol) | Residue | ΔGcalc (kcal/mol) |  |  |  |  |
| Val1 | -0.35 ± 0.30 | Arg3 | -1.30 ± 0.68 |  |  |  |  |
| Asp2 | 0.50 ± 0.12 | Arg5 | -8.13 ± 1.70 |  |  |  |  |
| Lys4 | 0.27 ± 1.07 | Leu9 | -1.86 ± 0.45 |  |  |  |  |
| **Lys5** | **-2.28 ± 1.18** | Glu13 | -0.65 ± 0.85 |  |  |  |  |
| Ala6 | -0.02 ± 0.07 | Leu50 | -3.40 ± 0.66 |  |  |  |  |
| Lys7 | 0.05 ± 0.11 | Thr53 | -0.51 ± 0.30 |  |  |  |  |
| **Phe8** | **-7.27 ± 0.99** | Val57 | -0.43 ± 0.35 |  |  |  |  |
| **His9** | **-1.94 ± 1.00** |  |  |  |  |  |  |
| Glu11 | 0.32 ± 0.34 |  |  |  |  |  |  |
| **Asp12** | **-1.34 ± 2.78** |  |  |  |  |  |  |
| **Tyr13** | **-1.48 ± 1.44** |  |  |  |  |  |  |

**SUPPLEMENTARY FIGURE LEGENDS**

**Supplementary Figure 1. Charge-charge interactions between peptide and ASC^PYD^.** The distance between the proton donors and acceptors of the side chains forming salt bridges is plotted against the simulation time. The salt-bridge partners are indicated in each panel, where the first residue corresponds to the peptide and the second residue to ASC^PYD^. In the case of Arg, Asp, and Glu, the distances are calculated from the midpoint of the following atoms: NH1 and NH2, OD1 and OD2, OE1 and OE2, respectively. For Lys, distances are calculated from the NZ atom.

**Supplementary Figure 2. Hydrophobic interactions between peptide and ASC^PYD^.** The distance between Phe10 or Ile8 of the peptide and the ASC^PYD^ hydrophobic pocket residues Arg5, Leu9 and Leu50 is plotted against the simulation time. For all four residues, distances are calculated from the midpoint of the side chain atoms starting from CB.

**Supplementary Figure 3. ms_ASC^PYD^ #2 as a negative control.** Docking simulations and residue-wise energy composition showing the limitation in binging for ms_ASC^PYD^ #2 compared to ms_ASC^PYD^ #4 and ms_ASC^PYD^ #5. (A) Docking simulations predicted a lower number of native-like conformations for ms_ASC^PYD^#2:ASC^PYD^ complex compared to ms_ASC^PYD^#5:ASC^PYD^ complex. Percentage of native like docked conformations (D1,D2 ≤ 5Å) are indicated in each case. (B) Residue-wise binding energy contributions for ms_ASC^PYD^ #2 show unfavorable (positive) energy contribution for Glu4. These data indicate that the negative charge at position 4 (Glu) is responsible to impede the binding of ms_ASC^PYD^ #2.

Fig. S1 (Pal et al.)

**
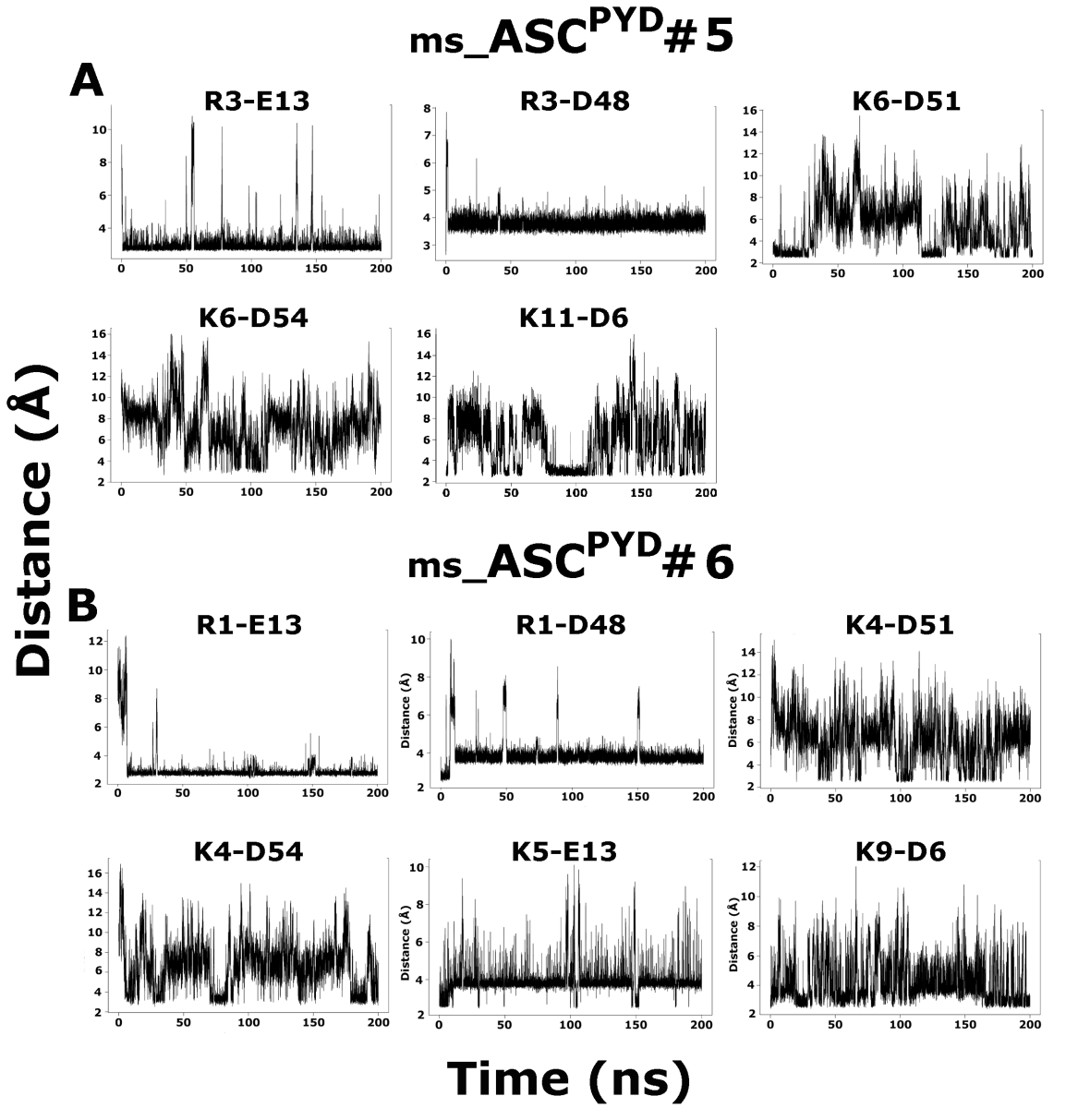
**

Fig. S2 (Pal et al.)


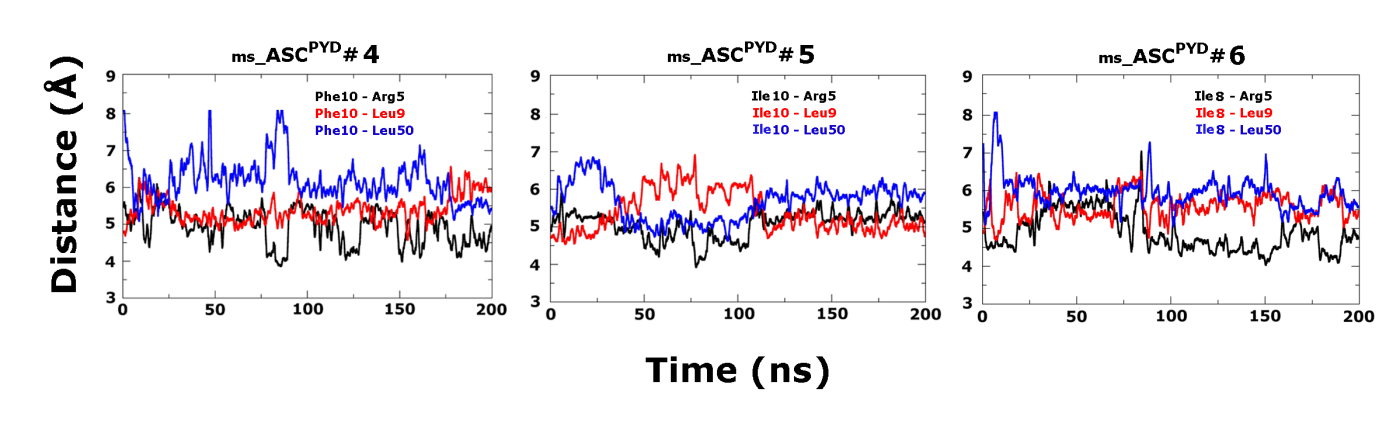


Fig. S3 (Pal et al.)


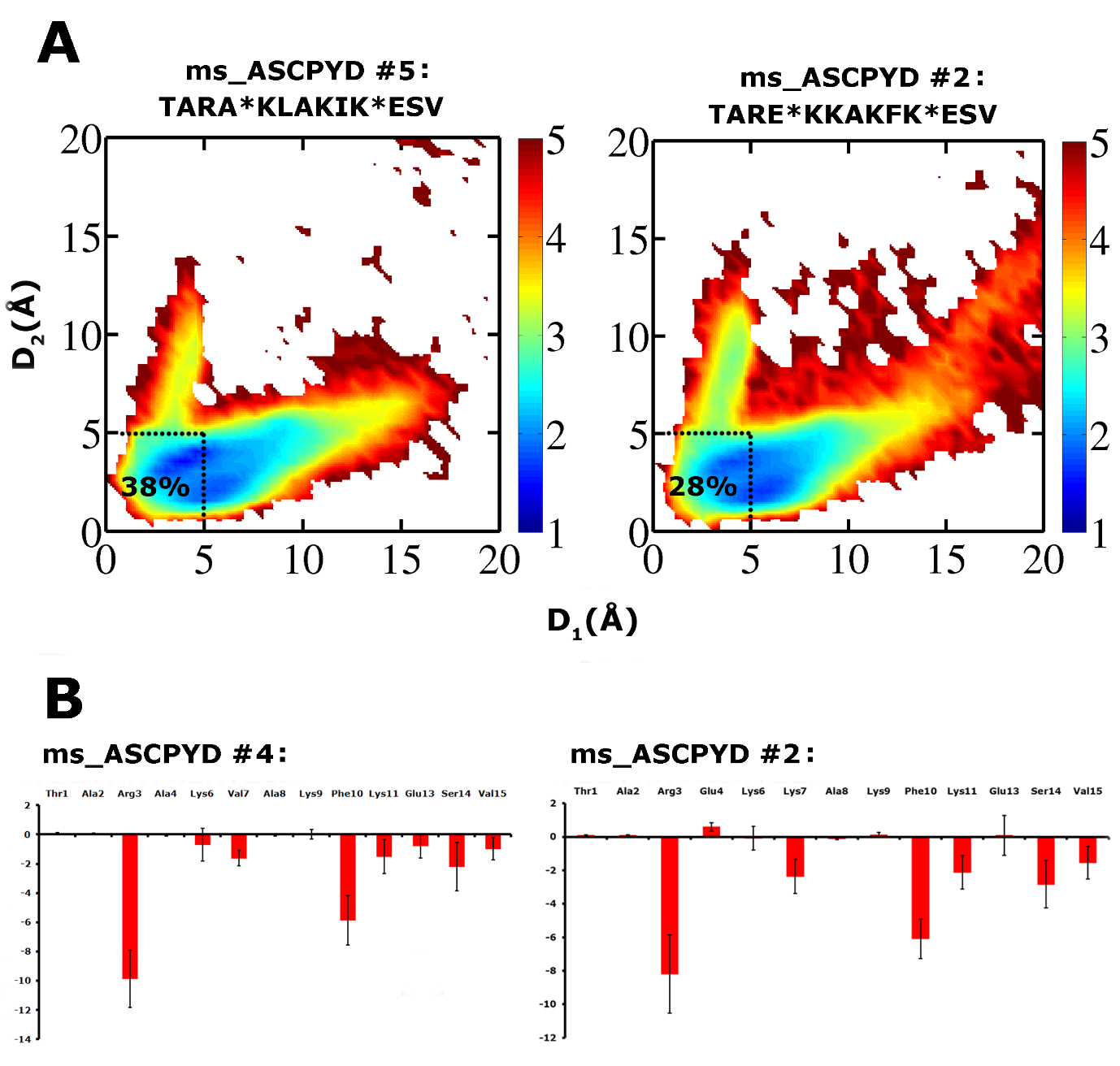

Supplement: Supplementary file 1 — Inhibition of NLRP3 inflammasome activation by cell-permeable stapled peptides [file 41598_2019_41211_MOESM1_ESM.docx]
